# Supplementary material for: The HBV basal core promoter mutation confers a replicative advantage and transcriptionally reprograms hepatocytes toward HCC subtypes
Source: bioRxiv. 2026 Sep 22:2026.09.22.752323. Preprint. [Version 1] doi: 10.64898/2026.09.22.752323 (PMC13622158; doi:10.64898/2026.09.22.752323)
Supplement: Supplementary file 1 [file media-1.pdf]

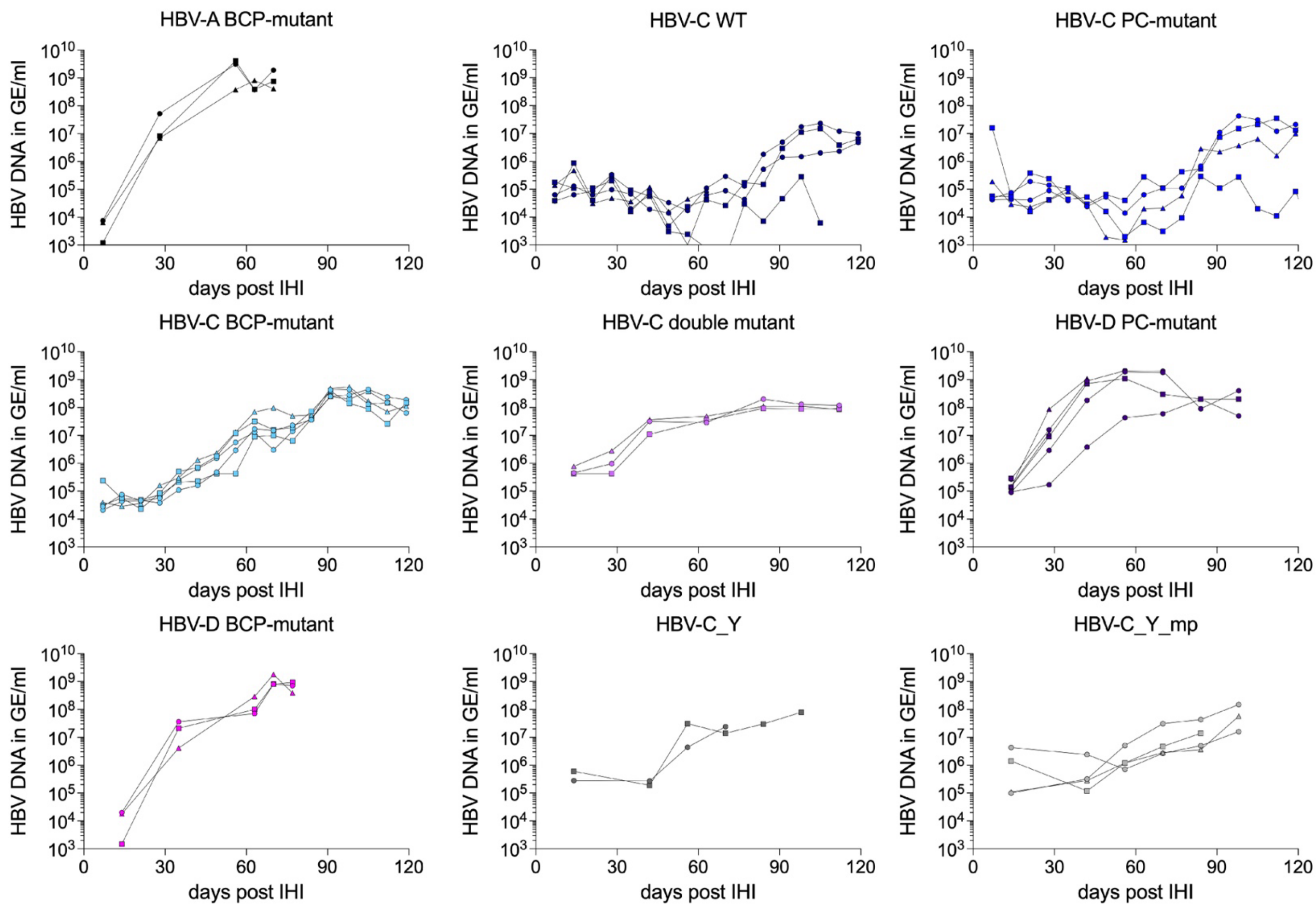

Figure S1: Successful launch of HBV strains and isogenic variants via IHI rcccDNA across genotypes A–D and patient consensus sequences.

Serum HBV viremia (HBV DNA in GE/ml) following IHI of rcccDNA in huFNRG mice is shown for nine independent HBV launches of WT strains and BCP- and PC-mutants across genotypes A–D. HBV_C_Y and HBV_CY_mp are patient-derived consensus sequences. *GE = genome equivalents, IHI = intrahepatic injection, rcccDNA = recombinant cccDNA.*

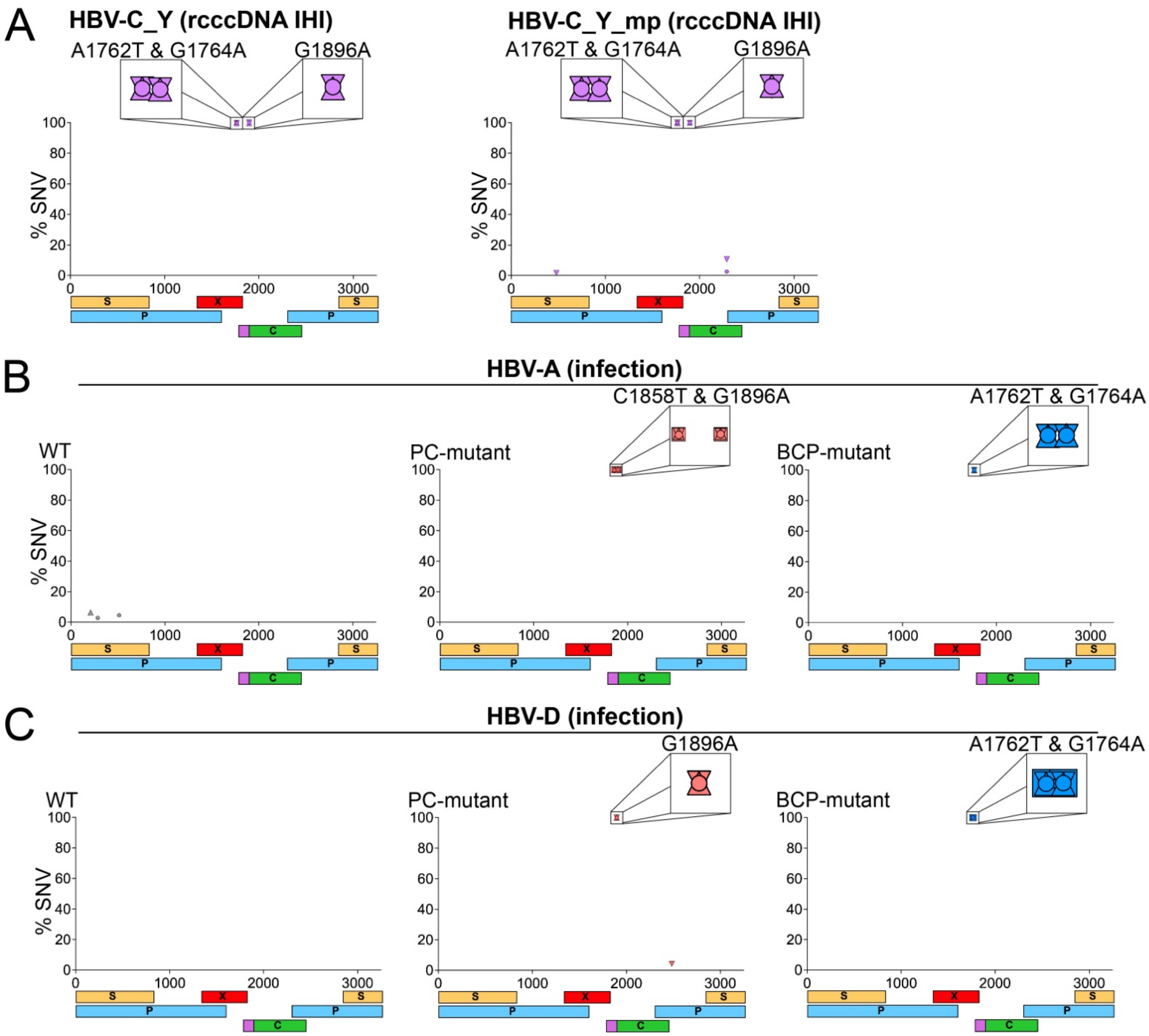

Figure S2: Viral sequencing of IHI rcccDNA launches and infection experiments.

(A) Sequencing analysis of HBV DNA isolated from serum of huFNRG mice following HBV rcccDNA IHI. Each data point represents an SNV, with each mouse represented by a different symbol (n= 3). The x-axis represents the position on the HBV genome, with the EcoRI restriction site in the S open reading frame (ORF) serving as reference starting point. S = S-antigen ORF, P = polymerase ORF, X = X-protein ORF, C = core ORF (the pink extension represents the ORF unique to the precore protein). **(B)** Sequencing analysis of HBV DNA isolated from serum of huFNRG mice following HBV-A infection. Each data point represents an SNV, with each mouse represented by a different symbol (n= 3-5). **(C)**

Sequencing analysis of HBV DNA isolated from serum of huFNRG mice following HBV-D infection. Each data point represents an SNV, with each mouse represented by a different symbol (n= 3-4). *huFNRG mice = humanized Fah$^{-/-}$ NOD Rag1$^{-/-}$ Il2rg^{null} mice, IHI = intrahepatic injection, rcccDNA = recombinant circular covalently close DNA, SNV = single nucleotide variation.*

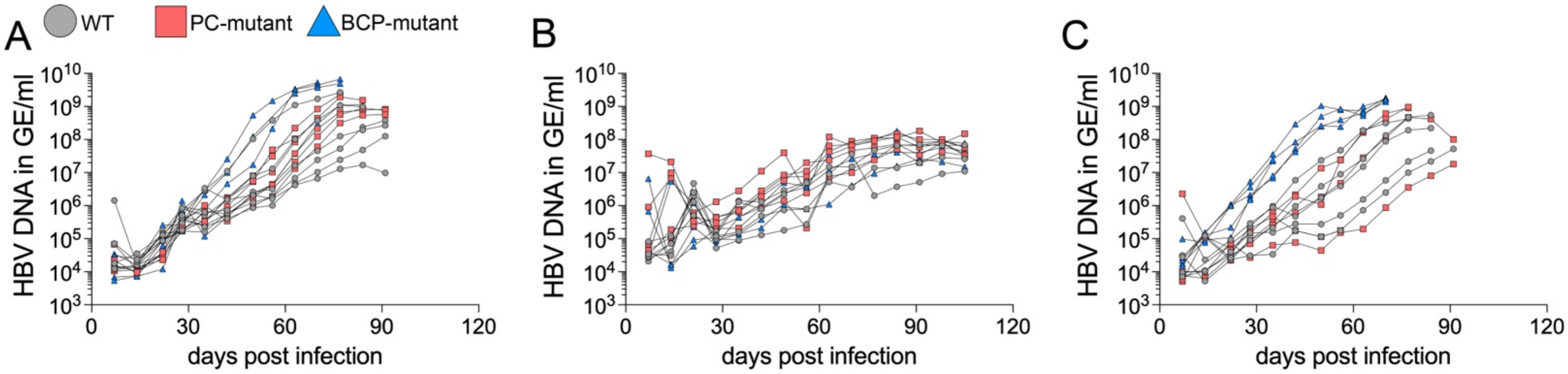

Figure S3: Hepatitis B infection with isogenic variants in HBV-A, HBV-C, and HBV-D: single mouse viremia.

*Related to **Fig. 2A**.* Viremia data from individual mice are presented. **(A)** HBV-A infection, **(B)** HBV-C infection, and **(C)** HBV-D infection. Mice were sacrificed at their individual peak plateau viremia level.

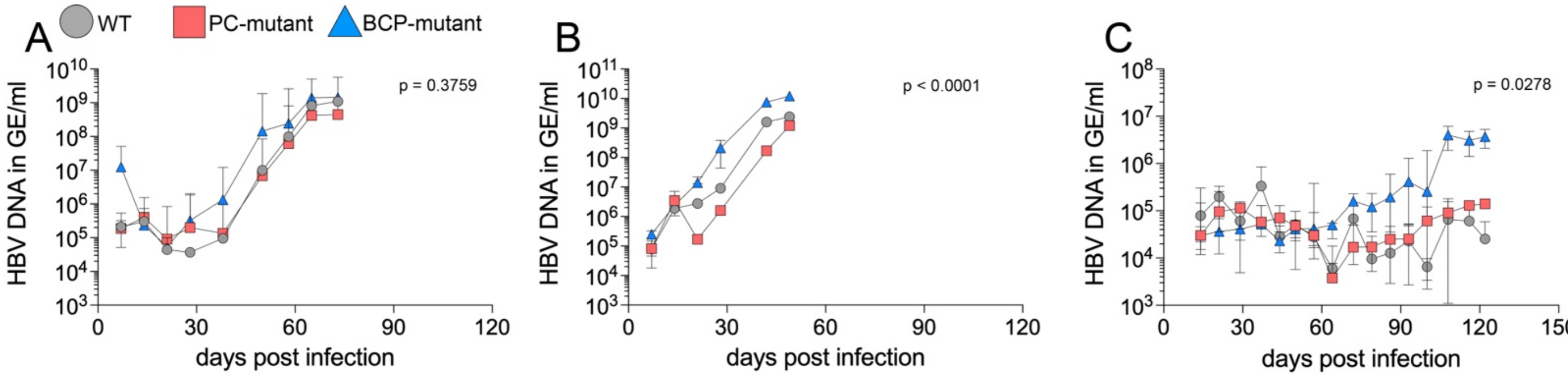

Figure S4: BCP-mutant infection consistently accelerates viremia kinetics in HBV-D infection. Independent infection experiments were performed to validate the observed differences in viral kinetics between WT, PC-, and BCP-mutant infection in HBV-D. HBV infections in **(A)** and **(B)** were performed in huFNRG mice engrafted with the same donor as in **Figure 2,** but in independent cohorts. Infections in **(C)** were performed in huFNRG mice engrafted with a different donor, resulting in lower human hepatocyte engraftment and serum human albumin levels (WT cohort: 1.66 mg/ml, range 0.26 - 6.51; PC-mutant cohort: 1.34 mg/ml, range 0.27 - 9.38; BCP-mutant cohort: 1.34 mg/ml, range 0.32 - 6.74). Data are shown as median values per group with interquartile range. Statistical analysis was performed using a linear mixed-effects model fitted by restricted maximum likelihood (REML), including fixed effects for *group*, *time*, and *group* × *time* interaction and a random effect for subject. **(A)** *group*: p = 0.0420; *time*: p = 0.1466; ***group* × *time*: p = 0.3759**, **(B)** *group*: p < 0.0001; *time*: p < 0.0001; ***group* × *time*: p < 0.0001**, **(C)** *group*: p = 0.0481; *time*: p = 0.0003; ***group* × *time*: p = 0.0278**. *GE = genome equivalents, huFNRG mice = humanized Fah$^{-/-}$ NOD Rag1$^{-/-}$ Il2rg^{null} mice*.

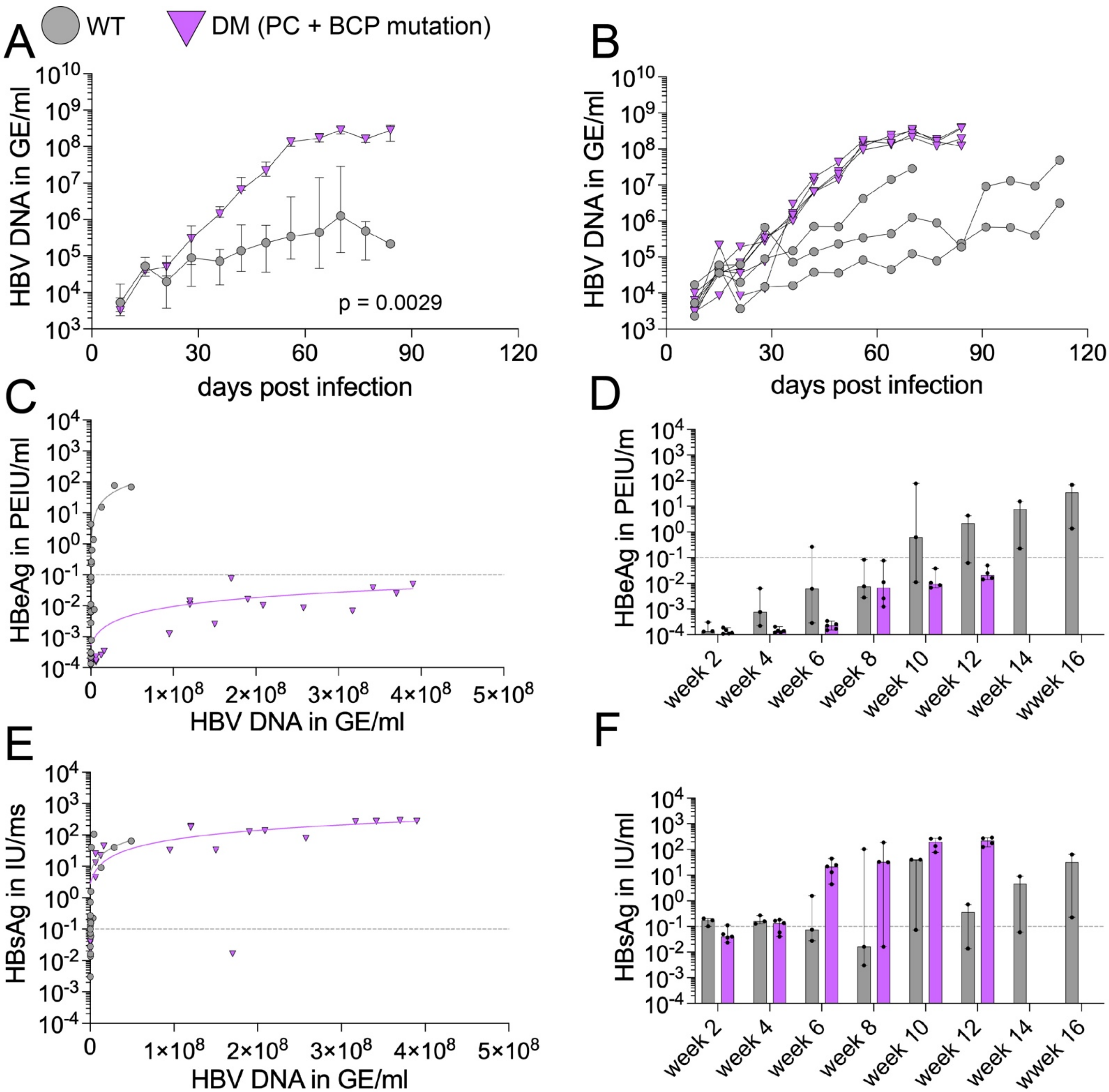

Figure S5: The double-mutant variant carrying both the PC- and BCP-mutation accelerates viral kinetics in HBV-C.

Highly humanized huFNRG mice (serum human albumin ~10 mg/mL) were infected with 10^5 GE via retroorbital injection with either WT (n=3) or double-mutant (DM) (n=6) HBV-C. Serum was collected weekly. **(A)** Grouped serum HBV viremia over time. Data are shown as median with interquartile range. Statistical analysis was performed using a linear mixed-effects model fitted by restricted maximum

likelihood (REML), including fixed effects for *group*, *time*, and *group* × *time* interaction and a random effect for subject. (*time*: $p = 0.0023$; *group*: $p < 0.0001$; ***group × time*: p = 0.0029**). **(B)** Serum viremia from individual mice. **(C)** Serum HBeAg levels, measured by chemiluminescence immunoassay, plotted against serum HBV viremia. Each symbol represents a single time point and animal. **(D)** Serum HBeAg levels over time.

(E) Serum HBsAg levels, measured by chemiluminescence immunoassay, plotted against serum HBV viremia. Each symbol represents a single time point and animal. **(F)** Serum HBsAg levels over time. *GE = genome equivalent, huFNRG mice = humanized Fah$^{-/-}$ NOD Rag1$^{-/-}$ Il2rg^{null} mice, IU = international units, LOQ = lower limit of quantification, PEIU = Paul-Ehrlich-Institute units*.

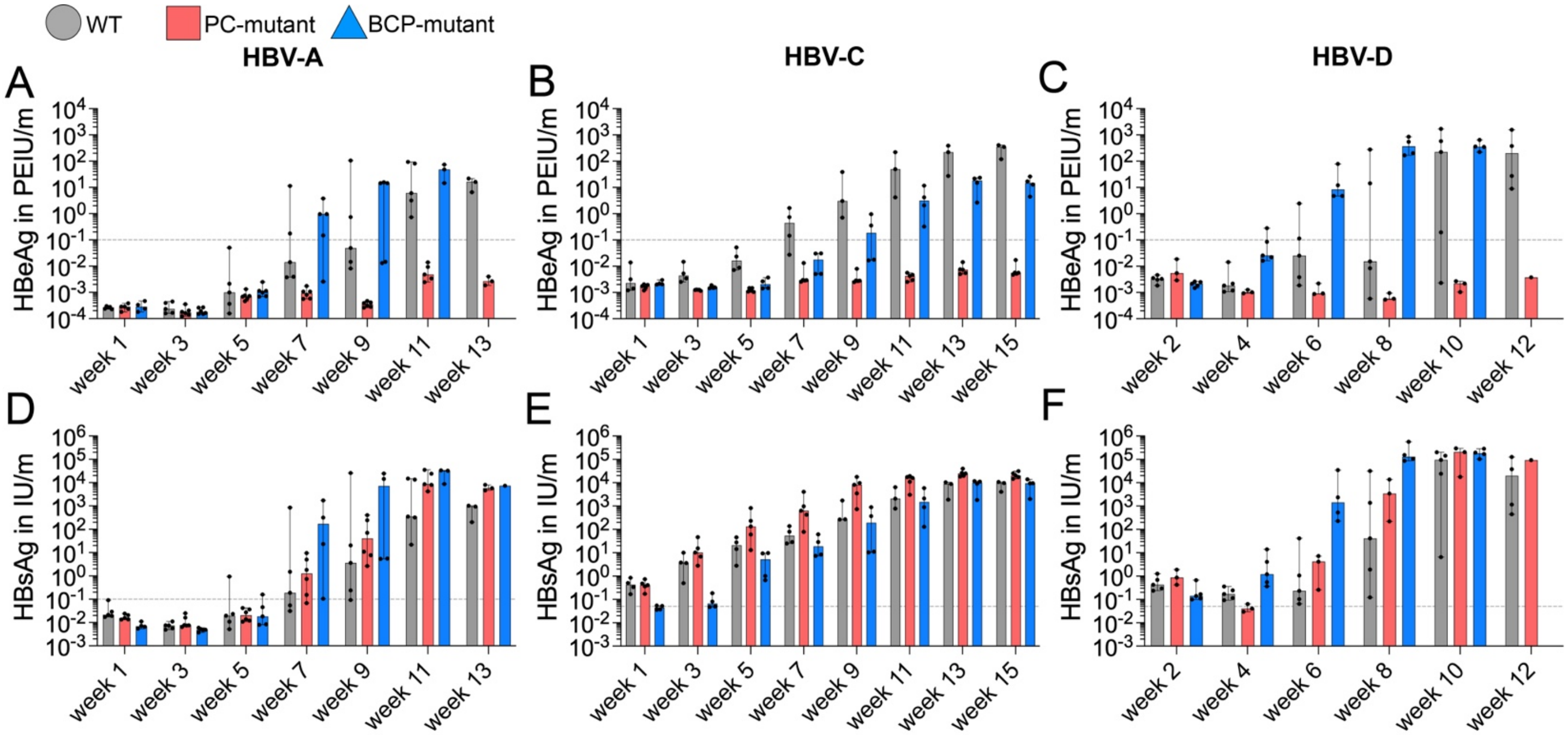

Figure S6: Time course of serum HBeAg and HBsAg levels in HBV-A, HBV-C, and HBV-D infection.

*Related to **Fig. 2B** and **2C***. **(A)** Serum HBeAg levels over time. **(B)** Serum HBsAg levels over time. *IU = international units, LOQ = lower limit of quantification, PEIU = Paul-Ehrlich-Institute units*.

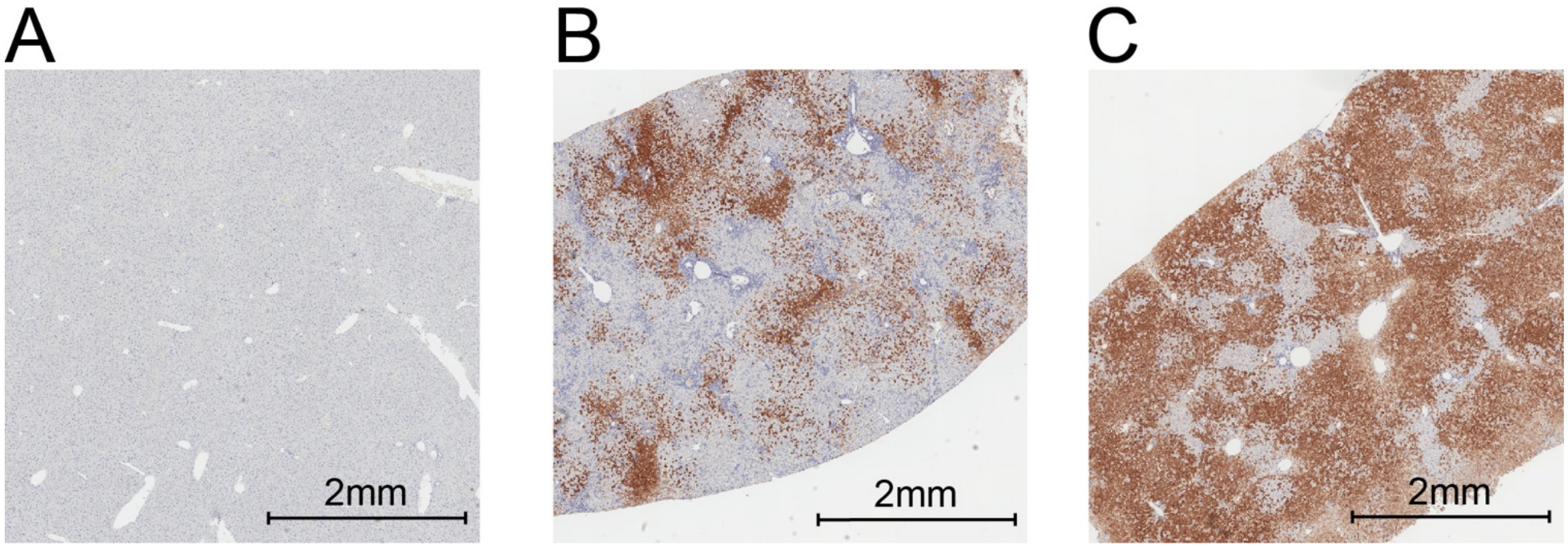

Figure S7: The human graft remains stable huFNRG mice with HBV infection.

Shown are representative images of FAH (absent in murine hepatocytes of FNRG mice) in **(A)** non-humanized, **(B)** lowly humanized (1mg/ml serum human albumin) or **(C)** highly humanized (10 mg/ml serum human albumin) huFNRG mice. FAH = fumarylacetoacetate hydrolase, *huFNRG mice* = *humanized* $Fah^{-/-}$ *NOD* $Rag1^{-/-}$ $Il2rg^{null}$ *mice*.

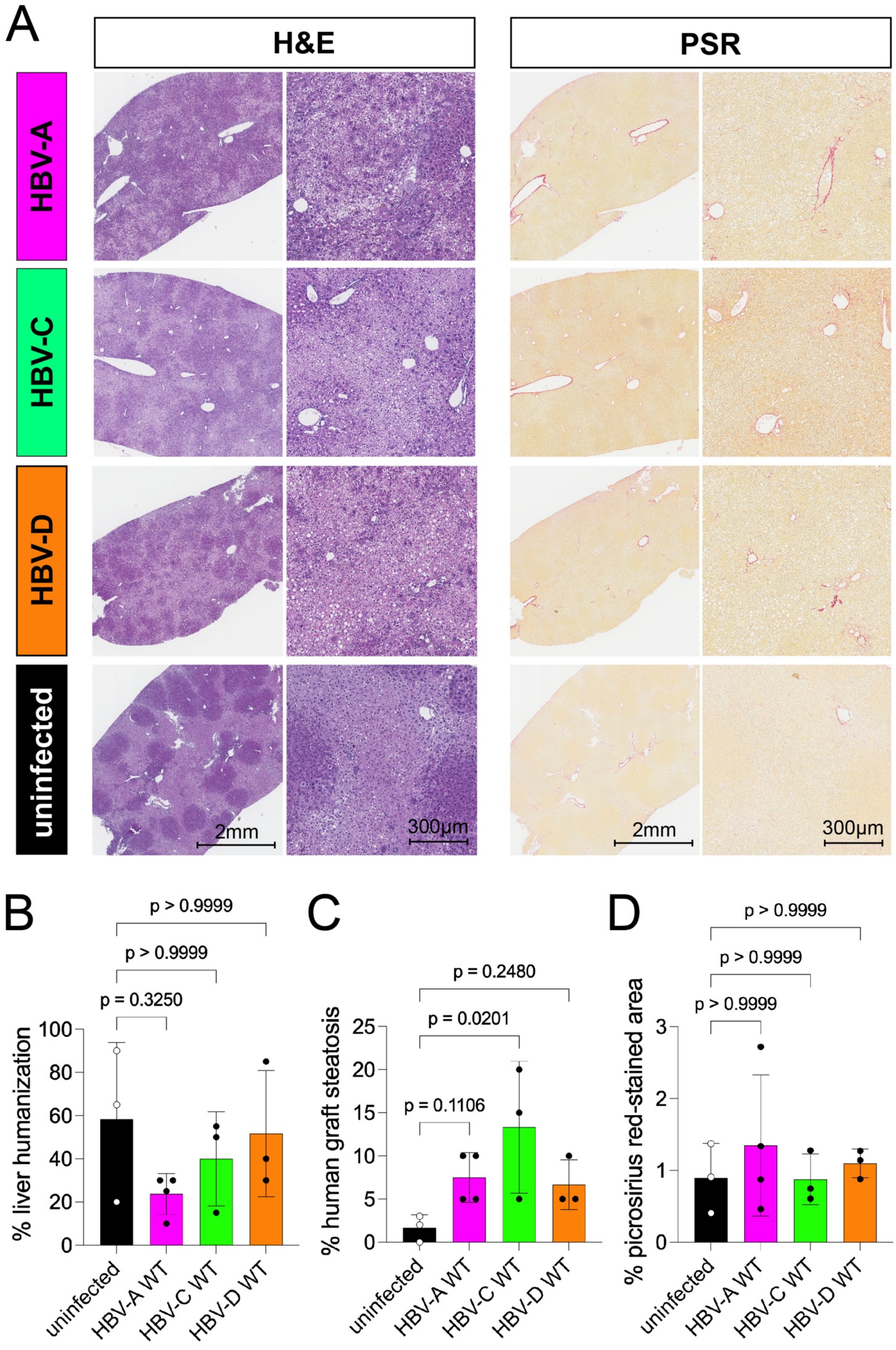
A
H&E
PSR
HBV-A
HBV-C
HBV-D
uninfected
2mm
300µm
2mm
300µm
B
p > 0.9999
p > 0.9999
p = 0.3250
% liver humanization
uninfected
HBV-A WT
HBV-C WT
HBV-D WT
C
p = 0.2480
p = 0.0201
p = 0.1106
% human graft steatosis
uninfected
HBV-A WT
HBV-C WT
HBV-D WT
D
p > 0.9999
p > 0.9999
p > 0.9999
% picrosirius red-stained area
uninfected
HBV-A WT
HBV-C WT
HBV-D WT

Figure S8: Liver histology in HBV WT infection.

(A) Representative H&E and PSR staining of liver sections from huFNRG mice infected with WT HBV-A, HBV-C, HBV-D, and uninfected controls. **(B)** Percentage of human hepatocytes among all hepatocytes. Statistics: Kruskal-Wallis test with Dunn's multiple comparisons test. **(C)** Percentage of steatosis in the human graft. Statistics: Kruskal-Wallis test with Dunn's multiple comparisons test. **(D)** Picrosirius red-stained area. Statistics: Kruskal-Wallis test with Dunn's multiple comparisons test. *huFNRG mice = humanized Fah*$^{-/-}$ *NOD Rag1*$^{-/-}$ *Il2rg*^{null} *mice*, *H&E = hematoxylin and eosin, PSR = picrosirius red stain*.

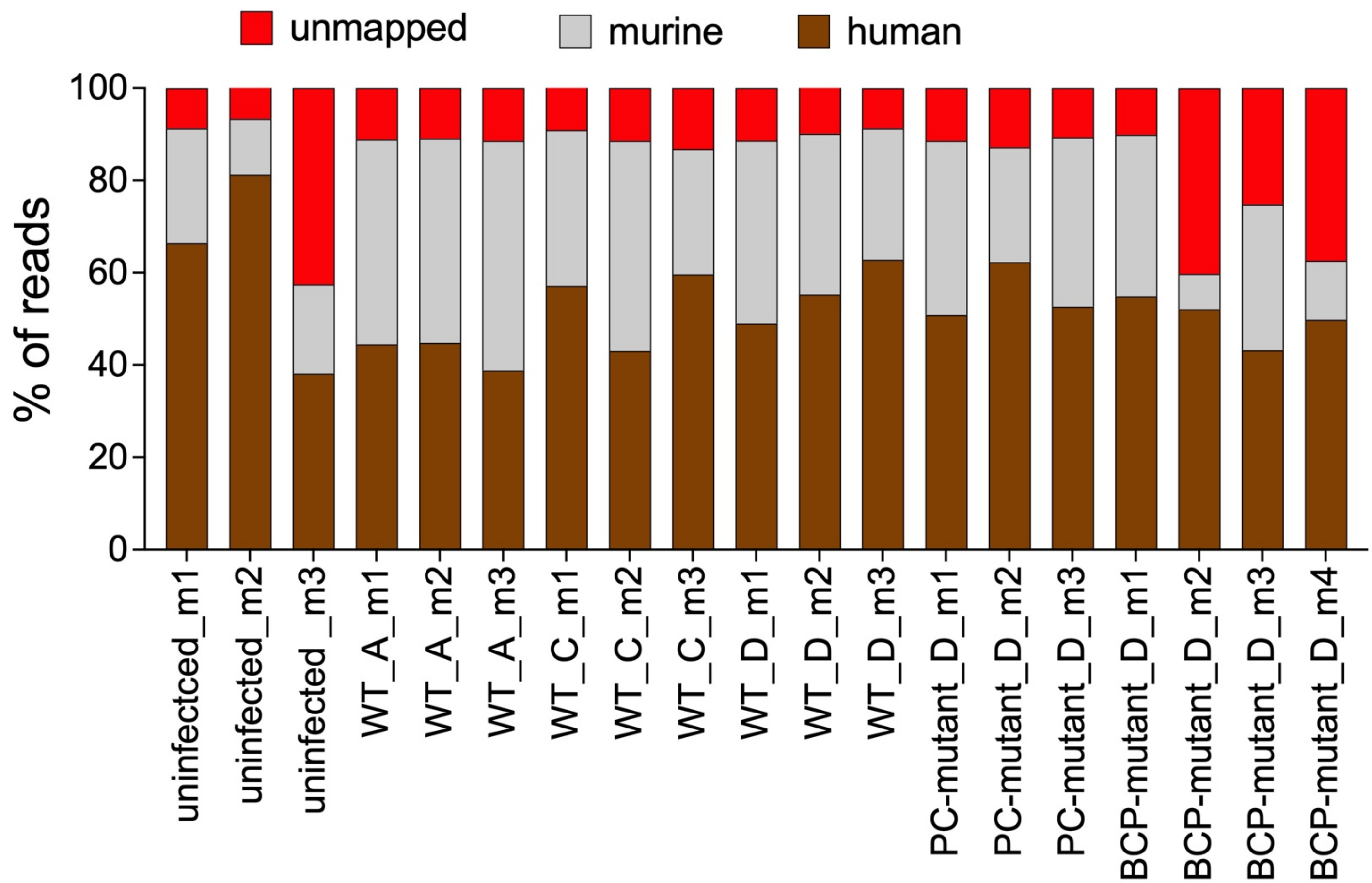

Figure S9: Proportion of human-, murine-, and ambiguously mapped reads in huFNRG-derived RNA.

Related to ***Fig. 3****:* Stacked bar plots show the percentage of sequencing reads assigned to the human (brown) or murine genome (grey) or unmapped/ambiguous reads (red) for each sample. Uninfected controls and HBV-infected human hepatocyte chimeric mice (HBV-A WT, HBV-C WT, HBV-D WT, HBV-D BCP-mutant, and HBV-D PC-mutant) are shown. *huFNRG mice = humanized Fah$^{-/-}$ NOD Rag1$^{-/-}$ Il2rg^{null} mice*.

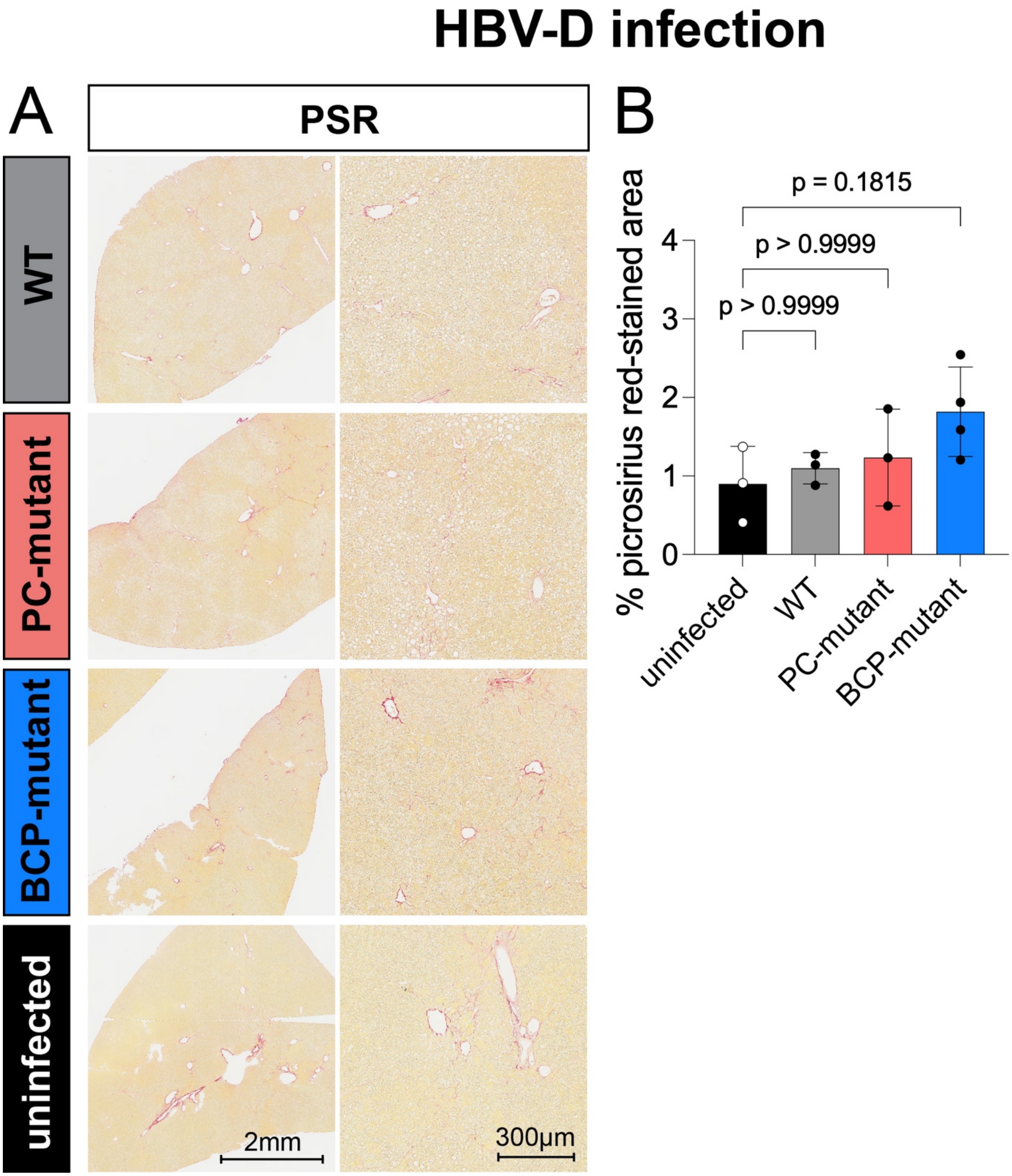

Figure S10: Picrosirius red-staining in HBV-D infection

Related to ***Fig. 4****:* **(A)** PSR staining of liver sections from huFNRG mice infected with HBV-D WT, BCP-mutant, PC-mutant, or uninfected controls*.* **(B)** Picrosirius red-stained area. Statistics: Kruskal-Wallis test with Dunn's multiple comparisons test. *huFNRG mice = humanized Fah$^{-/-}$ NOD Rag1$^{-/-}$ Il2rg^{null} mice, PSR = picrosirius red stain*.

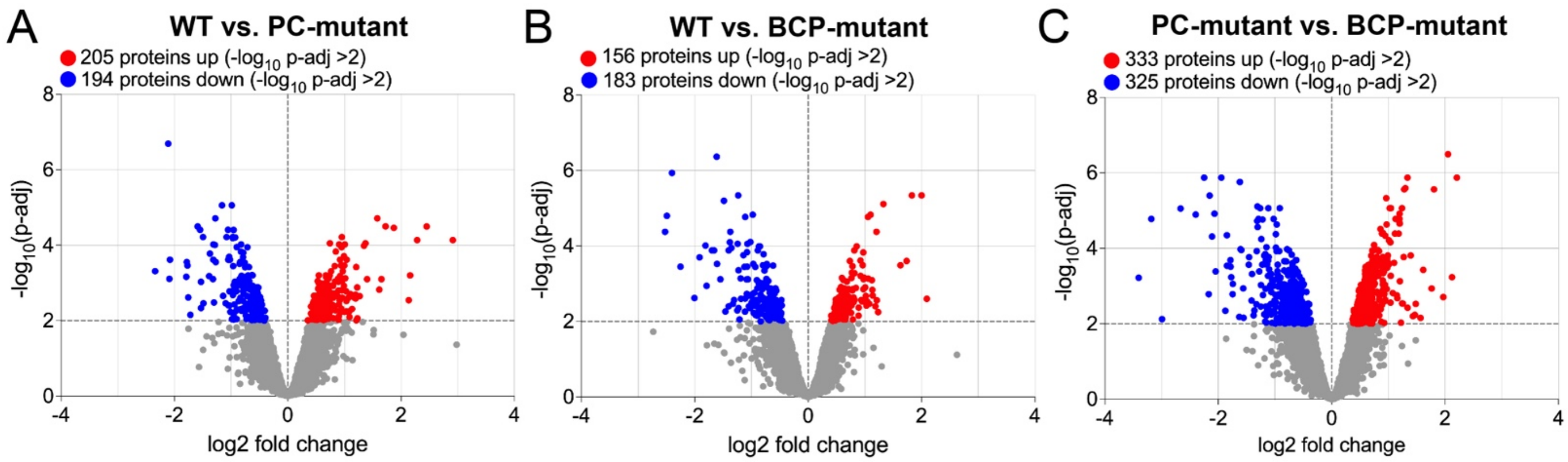

Figure S11: Volcano plots of differentially expressed proteins identified by SILAC-based proteomics in pre-infected mpPHHs.

*Related to **Fig. 5**:* Volcano plots show pairwise comparisons of protein abundance (heavy amino acid-labeled) between HBV variants: **(A)** WT vs. PC-mutant, **(B)** WT vs. BCP-mutant, and **(C)** PC- vs. BCP-mutant infection. *mpPHHs = mouse-passaged primary human hepatocytes.*

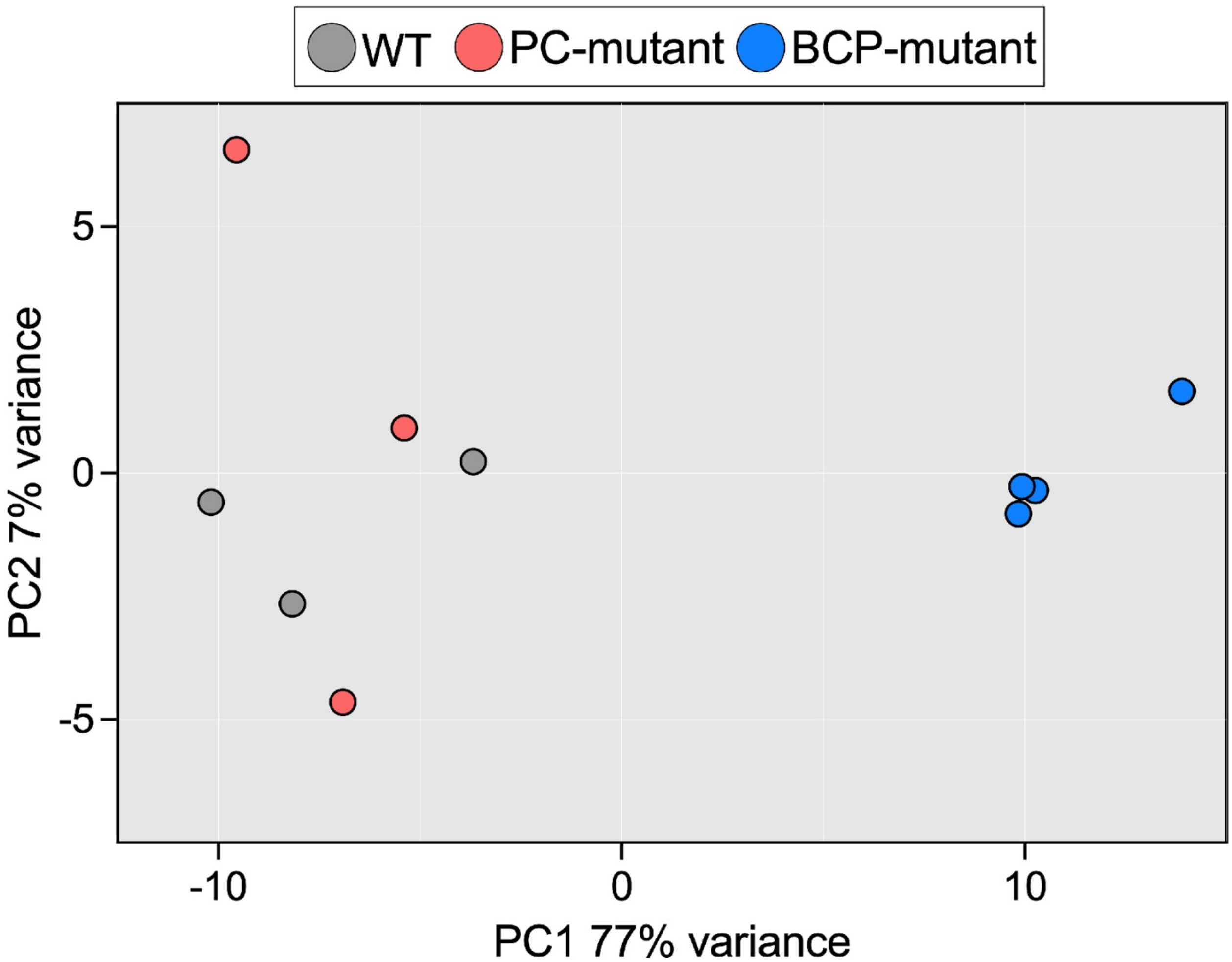

Figure S12: Principal component analysis of RNA-seq data from bulk liver tissue of huFNRG mice infected with HBV-D WT, BCP-mutant, or PC-mutant.

*Related to **Fig. 6**:* Principal component analysis based on the 1,000 most variable genes between HBV-D WT and BCP-mutant infected huFNRG mice.

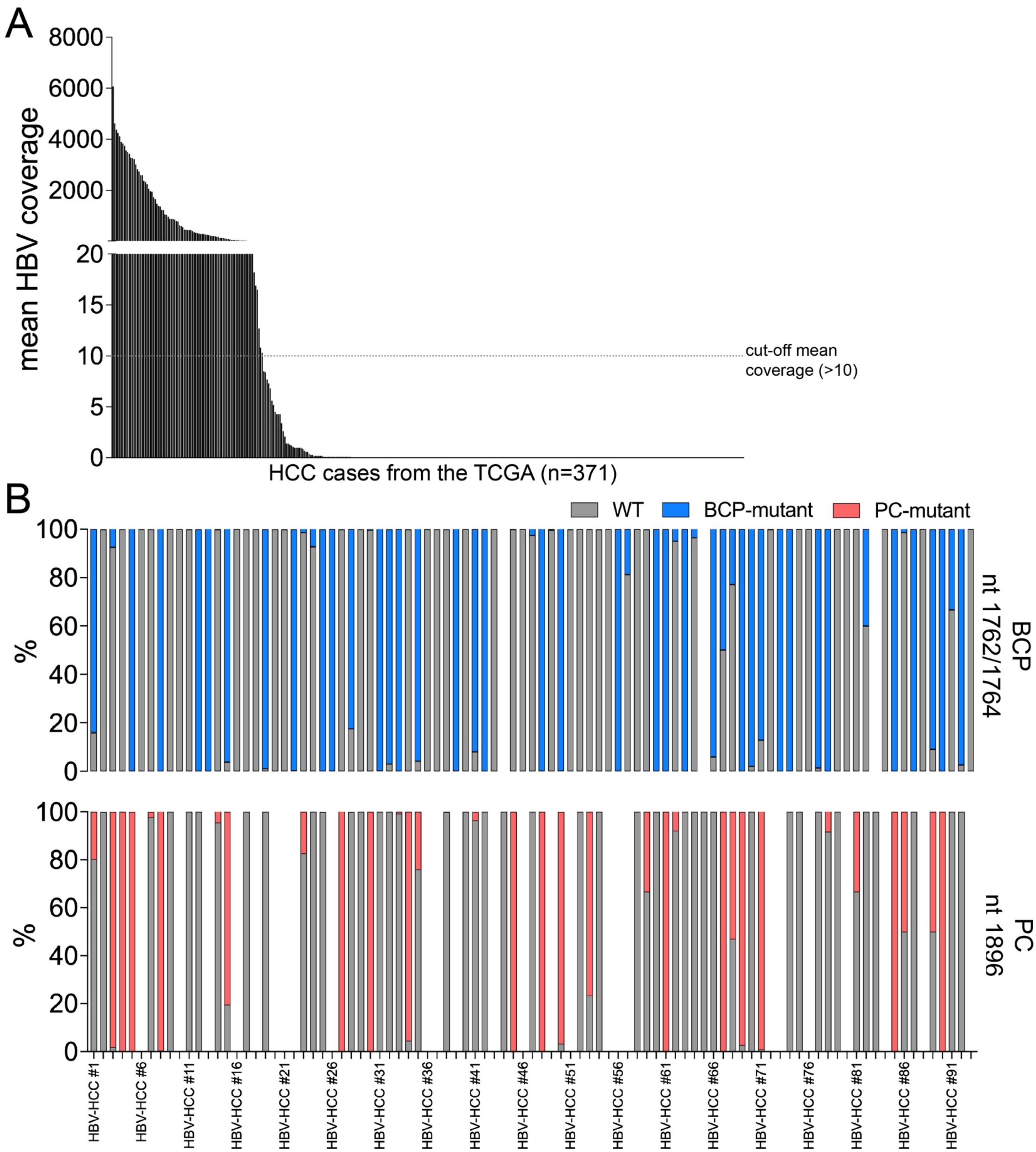

Figure S13: HBV sequencing coverage and relative variant abundance in human HCC. *Related to **Fig. 7***: All HCC cases in the TCGA were identified, and available RNA-seq data were accessed to identify HBV-derived RNAs. **(A)** Mean HBV coverage across all HCC cases (n = 371) from

TCGA. **(B)** Relative abundance of BCP- and PC-mutations across all HCC samples with detectable HBV RNA (HBV-HCCs) and a mean HBV coverage > 10. Cases are ordered by mean coverage as in **(A)**. *HCC = hepatocellular carcinoma, TCGA = The Cancer Genome Atlas.*

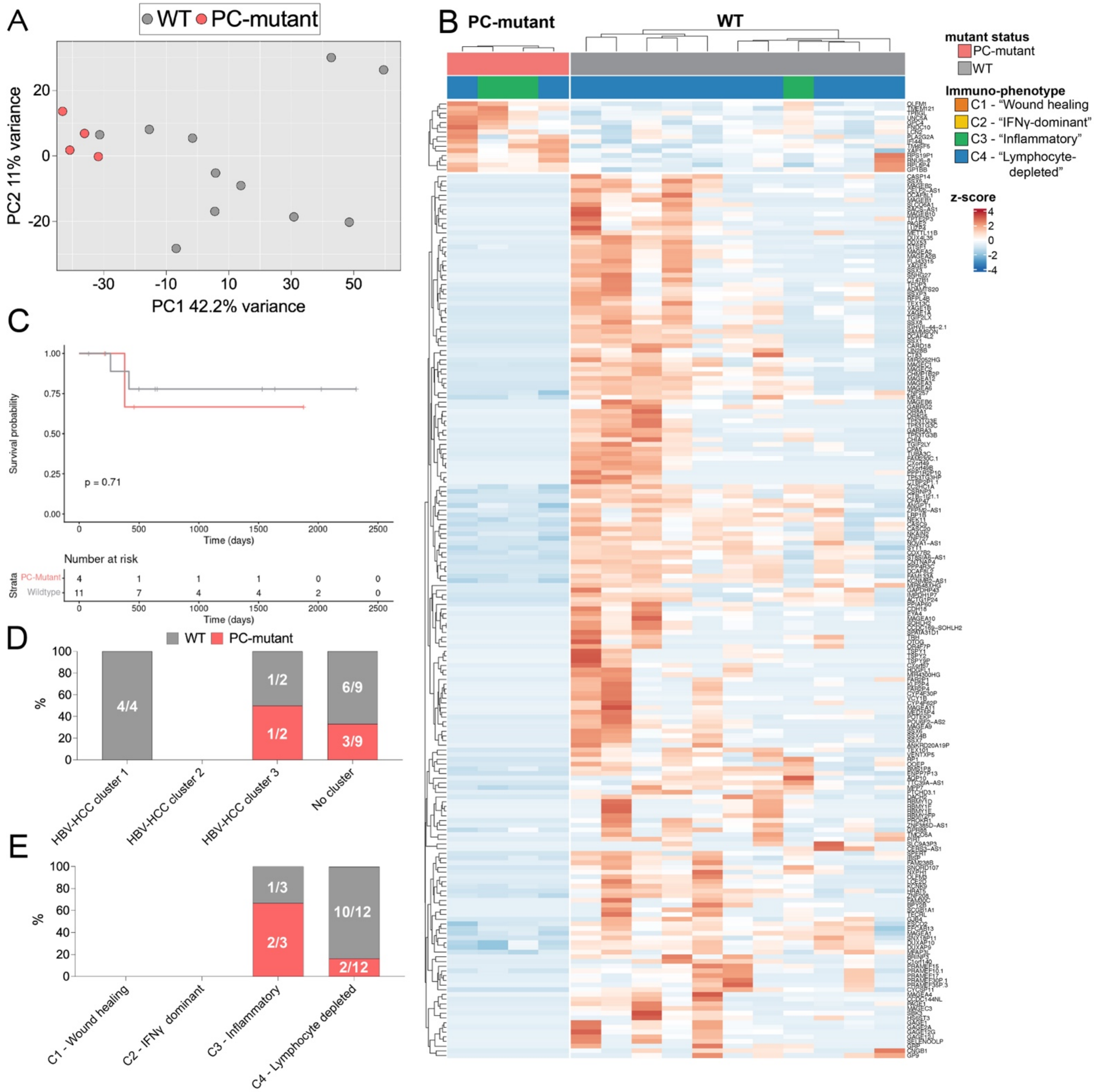

Figure S14: Comparison of HBV-associated HCCs from the TCGA dataset: **PC-mutant vs. WT.**

*Related to **Fig. 7***: HBV-HCC cases were classified as PC-mutant or WT if ≥ 97.5% of reads were PC-mutant or WT, respectively, at the PC locus nt1896, and samples with > 0 BCP-mutant reads, or coverage < 10 at the BCP loci nt1762/1764, were excluded ("pure" HBV-HCC samples). **(A)** Principal component analysis HBV-HCCs with WT or "pure" PC-mutant infection based on the 500 most variable genes. **(B)** Heatmap of differentially expressed genes between WT- and PC-mutant associated HCCs

(p-adj < 0.001, log2fc > |2|). **(C)** Kaplan-Meier survival analysis comparing WT- and PC-mutant associated HCCs. (p = 0.71, long-rank test). **(D and E)** Association of HBV WT- and PC-mutant associated HCCs with the HBV-HCC clusters from **(D)** Tian et al. or **(E)** immune subtypes. *fc = fold change, HCC = hepatocellular carcinoma, TCGA = The Cancer Genome Atlas.*

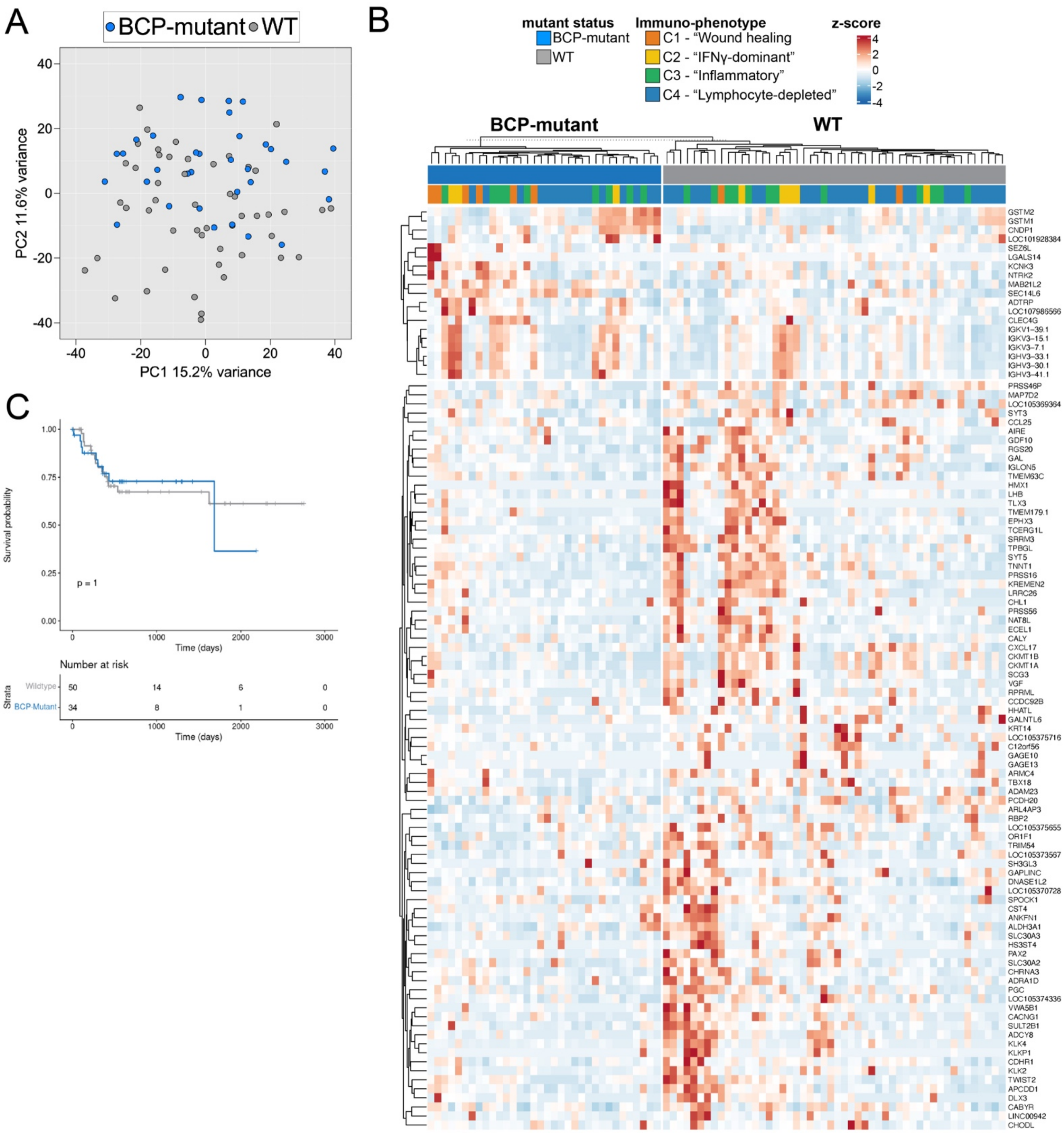

Figure S15: Comparison of HBV-associated HCCs from the TCGA dataset: BCP-mutant vs. WT ("majority call").

Related to ***Fig. 7***: HBV-HCC cases were classified as BCP-mutant or WT based on the majority of respective reads at position nt1762/1764 (≥ 50%), and samples with PC-mutation ≥ 50% were excluded ("majority call"). **(A)** Principal component analysis HBV-HCCs with WT or BCP-mutant infection based

on the 500 most variable genes. **(B)** Heatmap of differentially expressed genes between WT- and BCP -mutant associated HCCs (p-adj < 0.05, log2fc > $|2|$). **(C)** Kaplan-Meier survival analysis comparing WT- and BCP-mutant associated HCCs. (p = 1, long-rank test). *fc = fold change, HCC = hepatocellular carcinoma, TCGA = The Cancer Genome Atlas.*

Supplementary tables:

Table S1: HBV qPCR primer list

**isolate**	**fwd primer ID**	**fwd primer sequence**	**rev primer ID**	**rev primer sequence**	Taqman probe
**HBV-A**	RU-O-19375	CCGTCTGTGCCTTCTCATCTG	RU-O-19376	AGTCCAAGAGTCCTCTTATGTAAGACCTT	5- /56-FAM/CCG TGT GCA /ZEN/CTT CGCTTC ACCTCT GC/3IABkFQ/ -3
**HBV-B**	RU-O-33045	CCGTCTGTGCCTTCTCRTCTR	RU-O-32025	AGTCCAAGAGTCCKCTTATGYAAGACCTT	5- /56-FAM/CCG TGT GCA /ZEN/CTT CGCTTC ACCTCT GC/3IABkFQ/ -3
**HBV-C**	RU-O-32024	CCGTCTGTGCCTTCTCRTCTG	RU-O-32025	AGTCCAAGAGTCCKCTTATGYAAGACCTT	5- /56-FAM/CCG TGT GCA /ZEN/CTT CGCTTC ACCTCT GC/3IABkFQ/ -3
**HBV-D**	RU-O-19375	CCGTCTGTGCCTTCTCATCTG	RU-O-19376	AGTCCAAGAGTCCTCTTATGTAAGACCTT	5- /56-FAM/CCG TGT GCA /ZEN/CTT CGCTTC ACCTCT GC/3IABkFQ/ -3
**HBV-E**	RU-O-32024	CCGTCTGTGCCTTCTCRTCTG	RU-O-32025	AGTCCAAGAGTCCKCTTATGYAAGACCTT	5- /56-FAM/CCG TGT GCA /ZEN/CTT CGCTTC ACCTCT GC/3IABkFQ/ -3
**HBV-F**	RU-O-32024	CCGTCTGTGCCTTCTCRTCTG	RU-O-32025	AGTCCAAGAGTCCKCTTATGYAAGACCTT	5- /56-FAM/CCG TGT GCA /ZEN/CTT CGCTTC ACCTCT GC/3IABkFQ/ -3
**HBV-CY**	RU-O-32024	CCGTCTGTGCCTTCTCRTCTG	RU-O-32025	AGTCCAAGAGTCCKCTTATGYAAGACCTT	5- /56-FAM/CCG TGT GCA /ZEN/CTT CGCTTC ACCTCT GC/3IABkFQ/ -3

Table S2: HBV sequencing primer list

isolate	fwd primer ID	fwd primer sequence	rev primer ID	rev primer sequence
**HBV-A**	RU-O-32152	GCAACTTTTTCACCTCTGCCTAATCATC	RU-O-34056	CATGGTGCTGGTGCGCAG
**HBV-B**	RU-O-32152	GCAACTTTTTCACCTCTGCCTAATCATC	RU-O-34063	CATGGTGCTGGTGAACAC
**HBV-C**	RU-O-32152	GCAACTTTTTCACCTCTGCCTAATCATC	RU-O-34070	CATGGTGCTGGTGAACAG
**HBV-D**	RU-O-32152	GCAACTTTTTCACCTCTGCCTAATCATC	RU-O-34056	CATGGTGCTGGTGCGCAG
**HBV-E**	RU-O-32152	GCAACTTTTTCACCTCTGCCTAATCATC	RU-O-34056	CATGGTGCTGGTGCGCAG
**HBV-F**	RU-O-32152	GCAACTTTTTCACCTCTGCCTAATCATC	RU-O-34070	CATGGTGCTGGTGAACAG
**HBV-CY**	RU-O-32152	GCAACTTTTTCACCTCTGCCTAATCATC	RU-O-34070	CATGGTGCTGGTGAACAG

Table S3: Serum HBeAg levels in huFNRG mice

Group ID	n	Median $\Delta\log_{10}$	IQR (Q1–Q3)	Fold change (WT / BCP)	p value (Mann–Whitney U, two-sided)
**HBV-A WT**	5	–7.2389	–7.2889 to –7.2155	6.62× higher than BCP	—
**HBV-A BCP mutant**	4	–8.0598	–8.0839 to –7.9977	reference (1×)	0.0159
**Group ID**	**n**	**Median $\Delta\log_{10}$**	**IQR (Q1–Q3)**	**Fold change (WT / BCP)**	**p value (Mann–Whitney U, two-sided)**
**HBV-C WT**	3	–5.259	–5.864 to –5.065	32.82× higher than BCP	—
**HBV-C BCP mutant**	4	–6.775	–6.934 to –6.614	reference (1×)	0.0571
**Group ID**	**n**	**Median $\Delta\log_{10}$**	**IQR (Q1–Q3)**	**Fold change (WT / BCP)**	**p value (Mann–Whitney U, two-sided)**
**HBV-D WT**	5	–5.7694	–6.2245 to –5.6589	7.22× higher than BCP	—
**HBV-D BCP mutant**	4	–6.6279	–6.7213 to –6.5549	reference (1×)	0.0159
**Group (WT)**	**n**	**Median $\Delta\log_{10}$**	**IQR (Q1–Q3)**		
**HBV-A WT**	5	–7.2389	–7.2889 to –7.2155		
**HBV-C WT**	3	–5.2590	–5.8641 to –5.0651		
**HBV-D WT**	5	–5.7694	–6.2245 to –5.6589		
**A vs C (fold)**	**A vs C (p)**	**A vs D (fold)**	**A vs D (p)**	**C vs D (fold)**	**C vs D (p)**
0.010×	0.0357	0.034×	0.0079	—	—
95.5×	0.0357	—	—	3.24×	0.571
—	—	29.5×	0.0079	0.31×	0.571

Table S4: Serum HBsAg levels in huFNRG mice

**HBV-A**					
**Group**	**n**	**Median $\Delta\log_{10}$**	**IQR (Q1–Q3)**		
**WT**	19	–5.2893	–6.4418 to –5.0579		
**PC**	18	–5.0680	–6.1425 to –4.8236		
**BCP**	15	–5.4519	–5.7328 to –5.1863		
**WT vs PC (fold)**	**WT vs PC (p)**	**WT vs BCP (fold)**	**WT vs BCP (p)**	**PC vs BCP (fold)**	**PC vs BCP (p)**
0.6008×	0.2945	1.4543×	0.7287	—	—
1.6644×	0.2945	—	—	2.4205×	0.1335
—	—	0.6876×	0.7287	0.4131×	0.1335
**HBV-C**					
**Group**	**n**	**Median $\Delta\log_{10}$**	**IQR (Q1–Q3)**		
**WT**	28	–4.3578	–4.6788 to –3.8187		
**PC**	40	–3.8831	–4.2198 to –3.6210		
**BCP**	29	–4.6842	–5.1737 to –4.1535		
**WT vs PC (fold)**	**WT vs PC (p)**	**WT vs BCP (fold)**	**WT vs BCP (p)**	**PC vs BCP (fold)**	**PC vs BCP (p)**
0.34×	0.0718	2.12×	0.0487	—	—
2.98×	0.0718	—	—	6.33×	4.7×10^{-4}
—	—	0.47×	0.0487	0.16×	4.7×10^{-4}
**HBV-D**					
**Group**	**n**	**Median $\Delta\log_{10}$**	**IQR (Q1–Q3)**		
**WT**	34	–4.7428	–5.8355 to –3.6852		
**PC**	17	–3.8506	–5.4316 to –3.6495		
**BCP**	22	–5.1210	–5.8950 to –3.9136		
**WT vs PC (fold)**	**WT vs PC (p)**	**WT vs BCP (fold)**	**WT vs BCP (p)**	**PC vs BCP (fold)**	**PC vs BCP (p)**
0.13×	0.353	2.39×	0.551	—	—
7.80×	0.353	—	—	18.64×	0.098
—	—	0.42×	0.551	0.054×	0.098
**Group comparison**					
**group**	**n**	**Median $\Delta\log_{10}$**	**IQR (Q1–Q3)**		
**HBV-A WT**	19	–5.2893	–6.4418 to –5.0579		
**HBV-C WT**	28	–4.3578	–4.6788 to –3.8187		
**HBV-D WT**	34	–4.7428	–5.8355 to –3.6852		
**A vs C (fold)**	**A vs C (p)**	**A vs D (fold)**	**A vs D (p)**	**C vs D (fold)**	**C vs D (p)**
0.12×	0.0023	0.28×	0.018	—	—
8.54×	0.0023	—	—	2.43×	0.041
—	—	3.52×	0.018	0.41×	0.041

Table S5: Dysregulated KEGG pathways in SILAC proteomics in HBV-D infected mpPHHs

Table S5A: BCP-mutant vs. WT (up)				
**Enrichment FDR**	**nGenes**	**Pathway Genes**	**Fold Enrichment**	**Pathway**
2.14E-13	20	205	11.5769039	Path:hsa05203 Viral carcinogenesis
3.98E-12	18	190	11.241783	Path:hsa04613 Neutrophil extracellular trap formation
3.98E-12	16	138	13.7580597	Path:hsa05322 Systemic lupus erythematosus
4.79E-08	14	188	8.83662614	Path:hsa05034 Alcoholism
6.47E-07	14	234	7.0995116	Path:hsa05171 Coronavirus disease-COVID-19
3.76E-06	35	1556	2.66916085	Path:hsa01100 Metabolic pathways
5.61E-06	12	202	7.04930289	Path:hsa05169 Epstein-Barr virus infection
0.00010449	10	181	6.55598151	Path:hsa05168 Herpes simplex virus 1 infection
0.00042758	9	171	6.24543502	Path:hsa05164 Influenza A
0.00064212	6	69	10.3185448	Path:hsa05416 Viral myocarditis
0.00132008	8	159	5.97047876	Path:hsa04514 Cell adhesion molecules
0.00132008	6	80	8.8997449	Path:hsa04612 Antigen processing and presentation
0.00147825	9	210	5.08556851	Path:hsa05170 Human immunodeficiency virus 1 infection
0.00159783	6	86	8.27883246	Path:hsa04610 Complement and coagulation cascades
0.00299587	5	63	9.41771947	Path:hsa03250 Viral life cycle-HIV-1
0.00299587	7	139	5.97584789	Path:hsa05162 Measles
0.00368372	8	194	4.89333053	Path:hsa05167 Kaposi sarcoma-associated herpesvirus infection
0.00402308	4	38	12.49087	Path:hsa05330 Allograft rejection
0.00430661	7	153	5.42903828	Path:hsa01240 Biosynthesis of cofactors
0.00448777	5	72	8.24050454	Path:hsa04622 RIG-I-like receptor signaling pathway
0.0049155	7	159	5.22416891	Path:hsa05160 Hepatitis C
0.00532541	4	43	11.0384433	Path:hsa04940 Type I diabetes mellitus
0.00556661	4	44	10.7875696	Path:hsa05332 Graft-versus-host disease
0.00654217	5	82	7.23556496	Path:hsa04623 Cytosolic DNA-sensing pathway
0.00654217	8	223	4.25697813	Path:hsa05166 Human T-cell leukemia virus 1 infection
0.00741011	5	85	6.98019208	Path:hsa01232 Nucleotide metabolism
0.0096428	4	53	8.95571814	Path:hsa05320 Autoimmune thyroid disease
0.01882525	9	332	3.21677526	Path:hsa05165 Human papillomavirus infection
0.01894295	6	157	4.53490186	Path:hsa04218 Cellular senescence
0.0249792	7	224	3.70822704	Path:hsa05163 Human cytomegalovirus infection
0.04134813	5	131	4.52913226	Path:hsa04650 Natural killer cell mediated cytotoxicity

0.04254902	7	250	3.32257143	Path:hsa04144 Endocytosis
0.0454276	5	136	4.36262005	Path:hsa00190 Oxidative phosphorylation
**Table S5B: BCP-mutant vs. WT (down)**				
**Enrichment FDR**	**nGenes**	**Pathway Genes**	**Fold Enrichment**	**Pathway**
4.05E-07	12	170	10.5918786	Path:hsa04141 Protein processing in endoplasmic reticulum
1.51E-05	11	203	8.13087558	Path:hsa05205 Proteoglycans in cancer
2.12E-05	9	132	10.2307918	Path:hsa04142 Lysosome
2.88E-05	11	232	7.11451613	Path:hsa04820 Cytoskeleton in muscle cells
5.27E-05	10	202	7.42829767	Path:hsa04510 Focal adhesion
0.00119592	6	89	10.1158391	Path:hsa04512 ECM-receptor interaction
0.00121916	25	1556	2.41085496	Path:hsa01100 Metabolic pathways
0.00121916	7	138	7.6113137	Path:hsa04915 Estrogen signaling pathway
0.00447031	10	362	4.14507218	Path:hsa04151 PI3K-Akt signaling pathway
0.00709079	3	20	22.5077419	Path:hsa00100 Steroid biosynthesis
0.00709079	4	49	12.2491113	Path:hsa00330 Arginine and proline metabolism
0.01131209	6	152	5.92308998	Path:hsa04145 Phagosome
0.01197152	6	156	5.77121588	Path:hsa04148 Efferocytosis
0.01634125	7	231	4.54701857	Path:hsa04810 Reg. of actin cytoskeleton
0.0225609	4	75	8.00275269	Path:hsa01230 Biosynthesis of amino acids
0.0225609	2	9	33.3448029	Path:hsa03266 Virion-Herpesvirus
0.0225609	5	133	5.64103808	Path:hsa04270 Vascular smooth muscle contraction
0.0225609	4	80	7.50258065	Path:hsa04612 Antigen processing and presentation
0.0225609	5	129	5.81595399	Path:hsa04926 Relaxin signaling pathway
0.0225609	4	78	7.69495451	Path:hsa05100 Bacterial invasion of epithelial cells
0.0225609	7	248	4.23532778	Path:hsa05132 Salmonella infection
0.0225609	8	332	3.61570152	Path:hsa05165 Human papillomavirus infection
0.02499111	5	138	5.43665264	Path:hsa04910 Insulin signaling pathway
0.0295335	6	210	4.28718894	Path:hsa05170 Human immunodeficiency virus 1 infection
0.0296563	5	147	5.10379636	Path:hsa04072 Phospholipase D signaling pathway
0.0296563	3	44	10.2307918	Path:hsa04962 Vasopressin-regulated water reabsorption
0.03174898	4	93	6.45383281	Path:hsa04657 IL-17 signaling pathway
0.03322186	3	47	9.57776253	Path:hsa00514 Other types of O-glycan biosynthesis
0.03322186	6	224	4.01923963	Path:hsa05163 Human cytomegalovirus infection
0.03322186	4	97	6.18769538	Path:hsa05215 Prostate cancer

0.03349376	4	99	6.06269143	Path:hsa04933 AGE-RAGE signaling pathway in diabetic complications
0.03349376	4	99	6.06269143	Path:hsa05231 Choline metabolism in cancer
0.03610724	4	102	5.88437698	Path:hsa05146 Amoebiasis
0.03679179	6	236	3.81487151	Path:hsa04014 Ras signaling pathway
0.03797343	5	168	4.46582181	Path:hsa04140 Autophagy-animal
0.04651399	2	19	15.7949066	Path:hsa00531 Glycosaminoglycan degradation
0.04651399	4	115	5.21918654	Path:hsa01200 Carbon metabolism
0.04651399	4	115	5.21918654	Path:hsa04724 Glutamatergic synapse
0.04651399	5	181	4.14507218	Path:hsa05168 Herpes simplex virus 1 infection
**Table S5C: PC-mutant vs. WT (up)**				
**Enrichment FDR**	**nGenes**	**Pathway Genes**	**Fold Enrichment**	**Pathway**
5.00E-05	34	1556	2.61963003	Path:hsa01100 Metabolic pathways
0.00220768	8	136	7.05215282	Path:hsa00190 Oxidative phosphorylation
0.00321704	6	76	9.46473142	Path:hsa03320 PPAR signaling pathway
0.03555905	5	85	7.05215282	Path:hsa01232 Nucleotide metabolism
0.03555905	5	86	6.97015104	Path:hsa04610 Complement and coagulation cascades
0.04219503	5	93	6.44551602	Path:hsa04520 Adherens junction
**Table S5D: PC-mutant vs. WT (down)**				
**Enrichment FDR**	**nGenes**	**Pathway Genes**	**Fold Enrichment**	**Pathway**
1.98E-09	16	185	9.76457623	Path:hsa03040 Spliceosome
3.44E-05	9	103	9.86530304	Path:hsa03015 mRNA surveillance pathway
0.00050422	11	232	5.35315534	Path:hsa04820 Cytoskeleton in muscle cells
0.00119017	8	132	6.84260076	Path:hsa04142 Lysosome
0.00190532	7	107	7.38617185	Path:hsa03013 Nucleocytoplasmic transport
0.00473835	8	170	5.31307824	Path:hsa04141 Protein processing in endoplasmic reticulum
0.01037801	9	246	4.13059436	Path:hsa05131 Shigellosis
0.01985174	7	169	4.676452	Path:hsa04530 Tight junction
0.02369016	8	231	3.91005758	Path:hsa04810 Reg. of actin cytoskeleton
0.02369016	7	181	4.36641099	Path:hsa05168 Herpes simplex virus 1 infection
0.02564974	6	137	4.94465311	Path:hsa04210 Apoptosis
0.04273059	3	31	10.9260883	Path:hsa03060 Protein export

Table S6: KEGG pathways enriched in HBV-D BCP-mutant infected huFNRG mice (RNAseq)

Enrichment FDR	nGenes	Pathway Genes	Fold Enrichment	Pathway
1.51E-21	377	1556	1.63100793	Path:hsa01100 Metabolic pathways
4.37E-10	59	158	2.51373354	Path:hsa04110 Cell cycle
4.37E-10	139	529	1.7688192	Path:hsa05200 Pathways in cancer
2.37E-09	23	36	4.30080399	Path:hsa03030 DNA replication
4.85E-07	55	170	2.17790074	Path:hsa04141 Protein processing in endoplasmic reticulum
8.03E-05	45	149	2.0330617	Path:hsa05226 Gastric cancer
0.00017845	48	168	1.92334091	Path:hsa05225 Hepatocellular carcinoma
0.0002196	74	300	1.66048432	Path:hsa04010 MAPK signaling pathway
0.00066137	27	80	2.27194645	Path:hsa00983 Drug metabolism-other enzymes
0.00066137	33	107	2.07612968	Path:hsa04350 TGF-beta signaling pathway
0.00066137	105	478	1.47871922	Path:hsa05022 Pathways of neurodegeneration-multiple diseases
0.00076593	19	48	2.66462856	Path:hsa03272 Virion-Hepatitis viruses
0.00083479	12	23	3.51218776	Path:hsa03430 Mismatch repair
0.00087871	11	20	3.70243126	Path:hsa00100 Steroid biosynthesis
0.00113839	21	58	2.43733719	Path:hsa00480 Glutathione metabolism
0.00134417	31	103	2.02604358	Path:hsa04064 NF-kappa B signaling pathway
0.00180956	40	148	1.81937654	Path:hsa05224 Breast cancer
0.00193805	42	159	1.77818311	Path:hsa04217 Necroptosis
0.00193805	53	215	1.65944065	Path:hsa05207 Chemical carcinogenesis-receptor activation
0.0021437	26	84	2.08361932	Path:hsa04012 ErbB signaling pathway
0.0021437	59	248	1.60149153	Path:hsa05132 Salmonella infection
0.0021437	28	93	2.02674634	Path:hsa05222 Small cell lung cancer
0.0021437	38	141	1.81421519	Path:hsa05418 Fluid shear stress and atherosclerosis

0.0022984	37	137	1.8180485<br>3	Path:hsa04210 Apoptosis
0.0022984	41	157	1.7579581	Path:hsa04390 Hippo signaling pathway

Table S7: SILAC proteomics - PASEF acquisition table

#MS Type	Cycle Id	Start IM [1/K0]	End IM [1/K0]	Start Mass [m/z]	End Mass [m/z]	CE [eV]
MS1	0	-	-	-	-	-
PASEF	1	0.6	0.832	301.84	385.76	-
PASEF	1	0.832	1.5	621.8	631.29	-
PASEF	2	0.6	0.852	385.76	406.73	-
PASEF	2	0.852	1.5	631.29	640.98	-
PASEF	3	0.6	0.862	406.73	421.72	-
PASEF	3	0.862	1.5	640.98	650.82	-
PASEF	4	0.6	0.872	421.72	433.74	-
PASEF	4	0.872	1.5	650.82	660.83	-
PASEF	5	0.6	0.872	433.74	444.89	-
PASEF	5	0.872	1.5	660.83	670.84	-
PASEF	6	0.6	0.882	444.89	455.28	-
PASEF	6	0.882	1.5	670.84	681.02	-
PASEF	7	0.6	0.882	455.28	465.27	-
PASEF	7	0.882	1.5	681.02	691.88	-
PASEF	8	0.6	0.892	465.27	474.78	-
PASEF	8	0.892	1.5	691.88	703.33	-
PASEF	9	0.6	0.892	474.78	484.24	-
PASEF	9	0.892	1.5	703.33	715.38	-
PASEF	10	0.6	0.902	484.24	493.26	-
PASEF	10	0.902	1.5	715.38	727.37	-
PASEF	11	0.6	0.902	493.26	501.76	-
PASEF	11	0.902	1.5	727.37	740.4	-
PASEF	12	0.6	0.912	501.76	510.26	-
PASEF	12	0.912	1.5	740.4	754.03	-
PASEF	13	0.6	0.922	510.26	518.73	-
PASEF	13	0.922	1.5	754.03	768.36	-
PASEF	14	0.6	0.922	518.73	527.25	-
PASEF	14	0.922	1.5	768.36	782.91	-
PASEF	15	0.6	0.932	527.25	535.6	-
PASEF	15	0.932	1.5	782.91	798.44	-
PASEF	16	0.6	0.932	535.6	543.81	-
PASEF	16	0.932	1.5	798.44	815.91	-
PASEF	17	0.6	0.942	543.81	551.95	-
PASEF	17	0.942	1.5	815.91	833.42	-
PASEF	18	0.6	0.952	551.95	560.29	-
PASEF	18	0.952	1.5	833.42	853.44	-
PASEF	19	0.6	0.952	560.29	568.8	-
PASEF	19	0.952	1.5	853.44	875.73	-
PASEF	20	0.6	0.962	568.8	577.28	-

PASEF	20	0.962	1.5	875.73	900.93	-
PASEF	21	0.6	0.972	577.28	585.83	-
PASEF	21	0.972	1.5	900.93	928.51	-
PASEF	22	0.6	0.982	585.83	594.33	-
PASEF	22	0.982	1.5	928.51	961.97	-
PASEF	23	0.6	0.992	594.33	603.3	-
PASEF	23	0.992	1.5	961.97	1004.99	-
PASEF	24	0.6	1.012	603.3	612.32	-
PASEF	24	1.012	1.5	1004.99	1067.01	-
PASEF	25	0.6	1.052	612.32	621.8	-
PASEF	25	1.052	1.5	1067.01	1199.55	-

Table S8: SILAC proteomics - contrasts

RawfileName	SampleGroup
251209ko02LM_lab_sample01_1_1_6945.d	GTD_WT_noTreat
251209ko03LM_lab_sample02_1_1_6946.d	GTD_WT_noTreat
251209ko04LM_lab_sample03_1_1_6947.d	GTD_WT_noTreat
251209ko05LM_lab_sample04_1_1_6948.d	GTD_WT_IFNa
251209ko06LM_lab_sample05_1_1_6949.d	GTD_WT_IFNa
251209ko07LM_lab_sample06_1_1_6950.d	GTD_WT_IFNa
251209ko14LM_lab_sample13_1_1_6957.d	GTD_BCPM_noTreat
251209ko15LM_lab_sample14_1_1_6958.d	GTD_BCPM_noTreat
251209ko16LM_lab_sample15_1_1_6959.d	GTD_BCPM_noTreat
251209ko17LM_lab_sample16_1_1_6960.d	GTD_BCPM_IFNa
251209ko18LM_lab_sample17_1_1_6961.d	GTD_BCPM_IFNa
251209ko19LM_lab_sample18_1_1_6962.d	GTD_BCPM_IFNa
251209ko26LM_lab_sample25_1_1_6969.d	GTD_PCM_noTreat
251209ko27LM_lab_sample26_1_1_6970.d	GTD_PCM_noTreat
251209ko28LM_lab_sample27_1_1_6971.d	GTD_PCM_noTreat
251209ko29LM_lab_sample28_1_1_6972.d	GTD_PCM_IFNa
251209ko30LM_lab_sample29_1_1_6973.d	GTD_PCM_IFNa
251209ko31LM_lab_sample30_1_1_6974.d	GTD_PCM_IFNa
**Contrasts**	
GTD_WT_noTreat - GTD_WT_IFNa	-
GTD_PCM_noTreat - GTD_PCM_IFNa	-
GTD_BCPM_noTreat - GTD_BCPM_IFNa	-
GTD_WT_noTreat - GTD_PCM_noTreat	-
GTD_PCM_noTreat - GTD_BCPM_noTreat	-
GTD_WT_noTreat - GTD_BCPM_noTreat	-
